# Neighbourhood walkability and home neighbourhood-based physical activity: an observational study of adults with type 2 diabetes

**DOI:** 10.1186/s12889-016-3603-y

**Published:** 2016-09-09

**Authors:** Samantha Hajna, Yan Kestens, Stella S. Daskalopoulou, Lawrence Joseph, Benoit Thierry, Mark Sherman, Luc Trudeau, Rémi Rabasa-Lhoret, Leslie Meissner, Simon L. Bacon, Lise Gauvin, Nancy A. Ross, Kaberi Dasgupta, Diabetes, GPS, and Walkablilty Study Group, Sabiha Awan, Alexis Baass, Jean-Marie Boutin, Candace Lee, Carolina Capelle, Joanna Caron, Stavroula Christopolous, Karen Dahan, Maxine Dumas-Pilon, Roy Eappen, Leah Feldman, Natasha Garfield, Dominique Garrell, Lawrence Green, Walter Gregory, Wen Hu, Sofia Hussaini, John Hughes, Catherine Kudo, April Kinghorn, Evelyn Kyle, Leonora Lalla, Andrea Lalonde, Pierre Larochelle, Khue Ly, Richard Mackler, Agnieszka Majdan, Goldie Marmor, Sara Meltzer, Hortensia Mircescu, David Morris, Kimberly Munro, Vivian Petropoulos, Barry Posner, Brent Richards, Juan Rivera, Ghislaine Roederer, Samantha Sacks, Alicia Schiffrin, David Shannon, Raymond Sorge, Donald Sproule, Susan Still, Robert Wistaff, Mark Yaffe, Jean-Francois Yale, Hans Zingg

**Affiliations:** 1 Department of Epidemiology, Biostatistics and Occupational Health, McGill University, 1020 Pine Avenue West, Montreal, QC Canada; 2 Centre de Recherche du Centre Hospitalier de l’Université de Montréal (CHUM), Tour St-Antoine, S02-340, 850 St-Denis, Montreal, QC Canada; 3 Department of Medicine, Division of Internal Medicine, McGill University Health Centre, Montreal General Hospital, 1650 Cedar Avenue, C2.101.4, Montréal, QC Canada; 4 Division of Internal Medicine, Jewish General Hospital, 3755 Cote Sainte-Catherine, Montreal, QC Canada; 5 Department of Medicine, Division of Clinical Epidemiology, McGill University Health Centre, 687 Pine Avenue West, V1.08, Montréal, QC Canada; 6 Department of Medicine, Division of Endocrinology, McGill University Health Centre, 1001 Boulevard Decarie, Montreal, QC Canada; 7 Institut de Recherches Cliniques de Montréal (IRCM), 110 avenue des Pins, Montréal, QC Canada; 8 Department of Medicine, Division of Endocrinology, St. Mary’s Hospital, 3830 Lacombe Avenue, Montreal, QC Canada; 9 Montreal Behavioural Medicine Centre, Hôpital du Sacré-Cœur de Montréal, 5400 Boul. Gouin Ouest, Montréal, QC Canada; 10 Department of Exercise Science, Concordia University, 7141 Sherbrooke Street West, Montreal, QC Canada; 11 Département de médecine sociale et préventive, École de Santé Publique, Université de Montréal, 7101 avenue du Parc, Montréal, QC Canada; 12 Department of Geography, McGill University, 805 Sherbrooke Street West, Montreal, QC Canada

**Keywords:** Type 2 diabetes, Physical activity, Accelerometry, Global Positioning Systems, Physical activity locations, Neighbourhood walkability, Environmental epidemiology, Health geography

## Abstract

**Background:**

Converging international evidence suggests that diabetes incidence is lower among adults living in more walkable neighbourhoods. The association between walkability and physical activity (PA), the presumed mediator of this relationship, has not been carefully examined in adults with type 2 diabetes. We investigated the associations of walkability with total PA occurring within home neighbourhoods and overall PA, irrespective of location.

**Methods:**

Participants (*n* = 97; 59.5 ± 10.5 years) were recruited through clinics in Montreal (QC, Canada) and wore a GPS-accelerometer device for 7 days. Total PA was expressed as the total Vector of the Dynamic Body Acceleration. PA location was determined using a Global Positioning System (GPS) device (SIRF IV chip). Walkability (street connectivity, land use mix, population density) was assessed using Geographical Information Systems software. The cross-sectional associations between walkability and location-based PA were estimated using robust linear regressions adjusted for age, body mass index, sex, university education, season, car access, residential self-selection, and wear-time.

**Results:**

A one standard deviation (SD) increment in walkability was associated with 10.4 % of a SD increment in neighbourhood-based PA (95 % confidence interval (CI) 1.2, 19.7) – equivalent to 165 more steps/day (95 % 19, 312). Car access emerged as an important predictor of neighbourhood-based PA (Not having car access: 38.6 % of a SD increment in neighbourhood-based PA, 95 % CI 17.9, 59.3). Neither walkability nor car access were conclusively associated with overall PA.

**Conclusions:**

Higher neighbourhood walkability is associated with higher home neighbourhood-based PA but not with higher overall PA. Other factors will need to be leveraged to facilitate meaningful increases in overall PA among adults with type 2 diabetes.

**Electronic supplementary material:**

The online version of this article (doi:10.1186/s12889-016-3603-y) contains supplementary material, which is available to authorized users.

## Background

Adults with type 2 diabetes have low average levels of physical activity [1, 2]. Even modest increases may lead to important reductions in the risk for diabetes-related complications [3, 4]. It has been suggested that enhancing neighbourhood walkability may help facilitate increases in physical activity, particularly in older adults and/or in those living with chronic conditions [5–7].

Urban planners consider walkable neighbourhoods to be characterized by a variety of services and destinations easily accessed through well-connected street networks [8, 9]. These emerge when demand for services is high, as in more densely populated areas [10, 11]. Based on data from general adult populations, residents of such neighbourhoods report higher levels of utilitarian walking (e.g., walking to work) [12, 13]. There is a less consistent relationship between neighbourhood walkability and physical activity assessed objectively (i.e., with biosensor devices such as pedometers and accelerometers). While positive relationships have been delineated in Japan and in some European countries [14], the findings from North American studies are less clear [15, 16]. The relationship between neighbourhood walkability and physical activity has not been well-studied in type 2 diabetes, despite evidence of lower diabetes incidence in more walkable neighbourhoods [17, 18].

In the present study, we isolated the subset of total physical activity that occurs within home neighbourhoods and linked this to neighbourhood walkability in a cohort of adults with type 2 diabetes. We hypothesized that a relationship between neighbourhood walkability and physical activity would be more apparent if physical activity occurring specifically within neighbourhoods was considered. Two previous studies have investigated the relationship between neighbourhood walkability and home neighbourhood-based physical activity intensity in adults and demonstrated a positive relationship [19, 20]. We build on this work by examining total levels of physical activity occurring both within home neighbourhoods (excluding inside homes) and overall physical activity irrespective of location in adults with type 2 diabetes.

## Methods

### Participants and recruitment procedures

The study cohort was recruited between November 2012 and February 2015 during the baseline evaluations of an ongoing randomized controlled trial (Step Monitoring to Improve ARTERial Health, SMARTER; NCT0147520) [21]. The objective of SMARTER is to determine if physician-delivered step prescriptions lead to improvements in vascular disease risk among adults with type 2 diabetes or hypertension. Participants were ≥18 years of age at recruitment, under the care of a collaborating physician, and had a body mass index (BMI) between 25 and 40 kg/m^2^. Participants with co-morbid conditions that would impede accurate measurement of physical activity (e.g., visual impairments) or adherence to study procedures were excluded from the study. We enrolled SMARTER participants with a physician-diagnosis of type 2 diabetes and willing to wear an additional unit that combined an accelerometer with a GPS sensor for seven consecutive days as part of their baseline assessment. The SMARTER baseline assessment also included wearing a Yamax SW-701 pedometer with concealed viewing window for seven days. All participants provided written informed consent. Procedures were approved by McGill University’s Institutional Review Board (A08-M70-12B) and all participating institutions.

### Geographic Information System-derived neighbourhood walkability

Home neighbourhoods were approximated using 500-m polygonal street network buffers around home addresses using Geographic Information System (GIS) software (ArcMap 10.1; ESRI, Redlands, CA) and digital maps. *Street connectivity* within each buffer was computed as the number of ≥3-way intersections/km^2^. *Land use mix* was calculated using the entropy formula (−1) *Σ*_k_(p_k_lnp_k_)/ln N, where p represented the proportion of land area devoted to a specific land use (k) in each buffer and N represented the number of land uses that were being assessed (i.e., four; residential, commercial, institutional/governmental and recreational land uses). Street and land use files were obtained from DMTI CanMap Streetfiles [22]. *Population density* equaled the number of people per km^2^ of the census dissemination block where the home was located (2011 Canada Census Population Counts File). A walkability index was calculated by summing the z-scores of the street connectivity, land use mix, and population density measures. A higher index indicated greater walkability.

### Location-based physical activity

Physical activity and location were assessed with research-grade devices that integrate a GPS monitor (SIRF IV chip) and a tri-axial accelerometer (ADXL 345, Analog Devices) into one unit (96 × 80 × 31.80 mm, 125 g). Participants wore the GPS-accelerometer device on their hip for seven days during waking hours, except when showering, bathing, or swimming. They were instructed to connect their unit to a charger every night before going to bed. After the seven-day monitoring period, the device was mailed back to the research center in a postage-paid envelope. Physical activity was expressed as total Vector of the Dynamic Body Acceleration (VeDBA) accumulated over the total valid wear-period. Dynamic Body Acceleration correlates well with the rate of oxygen consumption [23, 24]. For the purposes of our study, VeDBA (i.e., the dynamic component of body acceleration (m/s^2^) integrated over a one-minute epoch) was summed over each participant’s valid wear time. In line with previously established methods [25, 26], we retained only individuals with four or more valid wear days (i.e., at least 10 h of valid data per day). Periods with one hour or more of consecutive accelerometer counts equal to zero were defined as non-wear time.

The GPS-accelerometer devices collected time-stamped latitudes and longitudes at 5-s intervals and raw accelerometer data at 50Hz on three axes. The location and accelerometer data were time-matched at the minute level. Participants’ homes were identified based on the density and distribution of GPS fixes using a ‘hot spot’ kernel-based detection algorithm [27]. Each hot-spot was verified to ensure that it matched the residential address that was provided by the participants. Participants with a mismatched home addresses were removed from the analyses. A spatial join was performed between the neighbourhood buffers and the GPS tracks of each participant to identify all GPS coordinates falling within the neighbourhood buffer but outside of the homes. Total VeDBA associated with these “inside neighbourhood” coordinates was computed.

### Pedometer-assessed daily steps

Daily steps were assessed for seven consecutive days at the baseline SMARTER evaluation (Yamax SW-701; viewing windows concealed). Participants were provided with two pedometers. Pedometer A was worn for seven consecutive days. Pedometer B remained in the postage-paid envelope and accounted for extra steps accumulated during the mailing process. Average daily steps were calculated as the number of steps accumulated on Pedometer A minus the number of steps accumulated on Pedometer B divided by the number of days the pedometer was worn. We created a robust linear regression model with which we established the relationship between the number of daily steps and the observed increments in VeDBA.

### Covariates

Season (spring/summer versus fall/winter) was defined based on the evaluation start date. Body mass index (BMI, kg/m^2^) was computed from weight and height measurements taken by a trained research assistant. The following were queried by questionnaire: age, sex, time since diabetes diagnosis, home address, married/common-law status, university education, employment, ethnicity, immigrant status, dog ownership, smoking status, insulin use, ownership and/or regular access to a motorized vehicle, depressed mood (Center for Epidemiologic Studies-Depression Scale score ≥16) [28], perceived neighbourhood walkability, and the importance of a neighbourhood’s walkability when choosing to move there.

### Statistical analyses

Descriptive statistics were produced, overall and by quartile of walkability. Associations between GIS-derived walkability and physical activity were assessed using robust linear regressions (m estimation with bisquare weighting) before and after adjustment for the following variables: age, BMI, sex, education, season, car access, residential self-selection and valid wear-time accumulated within neighbourhoods. Higher overall wear-time may allow an individual a greater opportunity to accumulate physical activity. Variables were retained based on theoretical importance and/or if they were identified based on correlation analyses (i.e., *R* ≥ 0.2) as potential confounders or predictors of neighbourhood-based physical activity. All variables were standardized so that the effect estimates of the linear regression models represented the percent change in 1-standard deviation (SD) of physical activity for a 1-SD increment in the GIS-derived walkability index. We approximated the number of pedometer-assessed daily steps associated with the observed increment in accelerometer-assessed VeDBA using robust linear regression models. To aid in the interpretation of our results, the relationship between BMI across quartiles of neighbourhood walkability was assessed using robust linear regressions. All statistical analyses were conducted using SAS 9.3 (SAS Institute Inc., Cary, NC, USA).

## Results

### Characteristics of study population

**Fig. 1:**
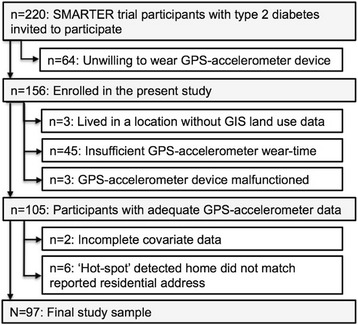
Selection of study cohort

Over 70 % of SMARTER participants eligible at the time of recruitment for this study agreed to participate (156/220) of whom 71.2 % had ≥ 4 valid wear days and 62.2 % had complete data on all variables of interest (Fig. 1). Most were married/common-law (69.1 %), university-educated (53.6 %), employed (61.9 %), and lived in the greater Montreal area (68.0 %). Just over half were men (56.7 %). The average age was 59.5 years (SD 10.5) and mean BMI was 31.5 kg/m^2^ (SD 4.5). On average, participants had diabetes diagnosis for 10.3 years (SD 7.6) and accumulated 4980 steps/day (SD 2798 steps/day). The rates of employment were similar among men (63.6 %) and women (59.5 %). VeDBA occurring anywhere was 615,687 (SD 240,065) and VeDBA occurring specifically within the residential neighbourhoods (excluding at home) was 26,113 (SD 39149).

Neighbourhoods had an average land use mix of 0.3 (SD 0.2), 27 three or more-way intersections/km^2^ (SD 14), and 8915 residents/km^2^ (SD 8351). Street connectivity, land use mix and population density increased across quartiles of neighbourhood walkability. The least walkable neighbourhoods had an average of 14 intersections per km^2^ (SD = 6), a land use mix of 0.04 (SD = 0.07), and a population density of 3920 people per km^2^ (SD = 2480). In Quartile 2 neighbourhoods there were 26 intersections/km^2^ (SD = 9), a land use mix of 0.21 (SD = 0.16), and population density of 4422 people/km^2^ (2421). In Quartile 3 neighbourhoods there were 30 intersections/km^2^ (SD = 8), a land use mix of 0.44 (SD = 0.15), and population density of 8462 people/km^2^ (SD = 5090). The most walkable neighbourhoods had an average of 37 intersections per km^2^ (SD = 18), a land use mix of 0.50 (SD = 0.17), and a population density of 18,621 people per km^2^ (9958). Neighbourhood walkability was moderate overall (average GIS-derived walkability score = 0, SD 2.15, Range: −3.5, 5.3). The least walkable neighbourhoods (Quartile 1 versus 4) had the highest proportions university education (70.8 % versus 52.0 %), dog owners (29.2 % versus 12.0 %), and participants with regular car access (91.7 % versus 64.0 %) (Table 1). No graded patterns were observed for VeDBA across quartiles of neighbourhood walkability. People living in the higher quartiles of neighbourhood walkability placed more importance on the walkability of a neighbourhood in selecting to move to a neighbourhood compared to people living in lower quartiles of neighbourhood walkability. Participants who were excluded from the final analyses (59/156) lived in less walkable neighbourhoods and included a larger proportion of women and a lower proportion of participants who were university educated, employed, immigrants, and/or had depressed mood (see Additional file 1). Those participants who were also excluded due to insufficient valid wear-time (i.e., 45 of these 59) included a larger proportion of individuals who had regular access to a car compared to those who were not excluded (*n* = 97) (i.e., 81.8 % versus 74.2 %) (See Additional file 1).

**Table 1:** Characteristics of study population (*n* = 97)

	Overall	Quartile of neighbourhood walkability^a^			
		Quartile 1	Quartile 2	Quartile 3	Quartile 4
	Mean (SD)	Mean (SD)	Mean (SD)	Mean (SD)	Mean (SD)
Age, *years*	59.5 (10.5)	60.6 (12.5)	58.8 (7.9)	57.7 (11.0)	60.6 (10.5)
Body mass index, *kg/m* ^*2*^	31.5 (4.5)	32.2 (4.9)	32.6 (4.9)	30.9 (4.0)	30.5 (3.9)
Time since diabetes diagnosis, *years*	10.3 (7.6)	9.9 (8.3)	9.6 (8.3)	10.8 (5.7)	11.1 (8.0)
Years at current residential address	18.9 (13.9)	22.9 (14.1)	15.9 (10.8)	20.1 (14.4)	17.0 (15.7)
Daily steps, *count*	4980 (2798)	4261 (1970)	5957 (3214)	4256 (2548)	5359 (3026)
Residential self-selection	0.001 (0.93)	−0.48 (0.71)	−0.12 (0.90)	0.06 (0.85)	0.53 (0.96)
Valid wear time, *days*	5.9 (1.0)	6.0 (1.0)	5.8 (1.1)	5.6 (0.9)	6.0 (1.1)
Total valid monitoring wear-time overall, *hours*	86.1 (21.1)	90.6 (21.0)	84.3 (21.8)	83.7 (20.3)	85.9 (21.7)
Total valid monitoring wear-time in neighbourhoods, *hours*	1.7 (2.8)	0.8 (0.5)	1.5 (1.3)	2.6 (5.3)	1.9 (1.5)
Time in neighbourhood, %	1.9 (2.4)	0.8 (0.6)	1.9 (2.1)	2.6 (3.7)	2.3 (2.0)
Total VeDBA					
Overall	615,687 (240,065)	601,822 (292,986)	619,913 (218,942)	611,492 (229,354)	628,632 (227,385)
In residential neighbourhoods (excluding home)	26,113 (39,149)	12,021 (13,781)	23,811 (27,747)	36,999 (67,888)	31,929 (24,518)
	*%*	*%*	*%*	*%*	*%*
Women	43.3	33.3	32.0	60.9	48.0
Married/common-law	69.1	70.8	68.0	73.9	64.0
University education	53.6	70.8	60.0	30.4	52.0
Employed	61.9	58.3	64.0	65.2	60.0
Immigrant	51.6	45.8	44.0	56.5	60.0
Depressed mood	30.9	29.2	16.0	43.5	36.0
Dog ownership	16.5	29.2	12.0	13.0	12.0
Ever smoker	44.3	54.2	48.0	26.1	48.0
Insulin use	30.9	33.3	20.0	26.1	44.0
Car access	74.2	91.7	80.0	60.9	64.0
Spring/summer assessment (versus fall/winter)	40.2	33.3	24.0	47.8	56.0

^a^Quartile cut-offs for the GIS-derived walkability index: Quartile 1: < −1.91 (*n* = 24); Quartile 2: ≥ − 1.91 < −0.04 (*n* = 25); Quartile 3: ≥ − 0.04 < 1.40 (*n* = 23); Quartile 4: ≥1.40 (*n* = 25); Neighbourhood walkability was based on polygonal-shaped buffers

### Multivariate analyses

Before and after adjustment for age, BMI and sex (Models 1 and 2), small but clinically important associations were observed between neighbourhood walkability and daily steps taken in home neighbourhoods. After further adjustment, these associations remained positive but included possibly clinically unimportant effects. In the fully adjusted model (Model 5) a 1-SD increment in walkability was associated with 10.4 % of a SD increment in neighbourhood-based physical activity (95 % confidence interval (CI) 1.2 to 19.7 %; Table 2). This would be similar to taking 165 more steps per day (95 % CI 19 to 312) within home neighbourhoods. No conclusive associations were observed between neighbourhood walkability and overall physical activity (i.e., that occurred anywhere; 0.7 %, 95 % CI −13.7 to 15.2 %; see Additional file 2).

**Table 2:** Regression estimates for the association between neighbourhood walkability and neighbourhood-based total VeDBA (*n* = 97)

	Percent change in one SD of total VeDBA (95 % confidence intervals)^a,b^	Corresponding change in daily steps (95 % confidence intervals)^c^
*Model 1*	21.2 (12.8 to 29.6)	337 (203 to 470)
*Model 2*	17.6 (9.3 to 26.0)	280 (148 to 413)
*Model 3*	13.9 (5.2 to 22.6)	221 (83 to 359)
*Model 4*	10.0 (0.7 to 19.3)	159 (11 to 307)
*Model 5*	10.4 (1.2 to 19.7)	165 (19 to 312)

^a^Model 1: Unadjusted. Model 2: Adjusted for age, BMI, sex. Model 3: Adjusted for age, BMI, sex, university, and season. Model 4: Adjusted for age, BMI, sex, university, season, car access and residential self-selection. Model 5: Adjusted for age, BMI, sex, university, season, car access, residential self-selection and valid wear-time

^b^Estimates represent the percent change in one standard deviation of total VeDBA (95 % confidence interval) occurring within home neighbourhoods (excluding homes) for every one-standard deviation increase in the GIS-derived neighbourhood walkability index. Calculated by multiplying the original estimate by the standard deviation of the walkability index (i.e., 2.16), dividing the result by the SD of the outcome (i.e., 39,149.22) and multiplying by 100

^c^Calculated using the following formula: daily steps = −548 + 0.0089*total VeDBA occurring anywhere)*(% change in one SD of VeDBA occurring in neighbourhood/100) where VeDBA occurring anywhere equals one SD of VeDBA occurring anywhere (i.e., 240,065.36)

Not having access to a car emerged as the strongest predictor of higher home neighbourhood-based physical activity after adjustment for factors identified a priori as potential confounders and covariates (Table 3). Those participants who did not have regular car access accumulated 38.5 % of a SD more in home neighbourhood-based physical activity (95 % CI 17.9, 59.3) compared to people who did have regular car access. This is equivalent to an increment of approximately 613 steps per day (95 % CI 284 to 942). No conclusive association was observed between car access and overall levels of physical activity (11.1 % of a SD increment in neighbourhood-based physical activity for participants with regular car access compared to participants without regular car access, 95 % CI −21.3 to 43.5).

**Table 3:** Regression estimates for the full-adjusted neighbourhood-based total VeDBA model with corresponding changes in daily steps (*n* = 97)^a^

	Percent change in one SD of total VeDBA (95 % confidence intervals)^b^	Corresponding change in daily steps (95 % confidence intervals)^c^
Age, *years*	−0.01 (−0.9 to 0.8)	−0.1 (−14 to 14)
Women	−8.5 (−26.5 to 9.4)	−135 (−421 to 150)
Body mass index, *kg/m* ^*2*^	−1.2 (−3.2 to 0.9)	−19 (−51 to 14)
University educated *(yes* versus *no)*	−8.8 (−26.8 to 9.1)	−140 (−425 to 144)
Spring/summer assessment *(*versus *fall/winter)*	16.1 (−1.2 to 33.4)	256 (−19 to 531)
Regular car access *(yes* versus *no)*	−38.6 (−59.3 to −17.9)	−613 (−942 to −284)
Residential self-selection score	5.3 (−4.5 to 15.1)	84 (−72 to 240)
Valid wear-time, *minutes*	0.01 (−0.002 to 0.01)	0.1 (−0.03 to 0.2)

^a^This fully adjusted model is additionally adjusted for GIS-derived neighbourhood walkability

^b^Effect estimates represent the percent change in one standard deviation of total VeDBA occurring in home neighbourhoods (excluding homes) for every one-unit increase in the predictor of interest. Calculated by dividing the original beta estimate by the standard deviation of VeDBA (occurring within home neighbourhoods but excluding homes) (i.e., 39,149.22) and multiplying by 100

^c^Calculated using the following formula: daily steps = −548 + 0.0089*total VeDBA occurring anywhere)*(% change in one SD of VeDBA occurring in neighbourhood/100) where VeDBA occurring anywhere equals one SD of VeDBA occurring anywhere (i.e., 240,065.36)

After adjustment for age, sex and education, there was a signaled but inconclusive association between neighbourhood walkability and BMI: Participants who lived in the most compared to the least walkable neighbourhoods (Quartile 4 versus Quartile 1) had a 1.6 kg/m^2^ decrement in BMI (95 % CI −4.1 to 0.9). This signaled association remained after further adjustment for total physical activity occurring anywhere (i.e., −1.5 kg/m^2^, 95 % CI −3.9 to 1.0).

## Discussion

Our study population achieved an average of 4980 steps/day, placing them in the “sedentary” category according to the cut-offs proposed by Tudor-Locke [29] and well below the recommended target of 10,000 steps per day [26]. This step count is consistent with the findings of previous studies of adults with type 2 diabetes [2, 7]. Improving neighbourhood walkability has been suggested as a means of facilitating increases in walking [5–7]. Our analyses demonstrate that higher neighbourhood walkability is associated with somewhat higher levels of neighbourhood-based physical activity in adults with type 2 diabetes after adjustment for age, BMI, sex, education, season, car access, and residential self-selection. There was no conclusive evidence, however, that individuals living in walkable neighbourhoods accumulated higher levels of overall physical activity (i.e., activity inside the neighbourhoods and elsewhere). Not having regular access to a car was the most important predictor of home neighbourhood-based physical activity.

These findings are consistent with our previous analysis of 2949 Canadian adults who participated in Cycle 1 of the Canadian Health Measures Survey [16], but in contrast to data from Europe and Asia. Our recent meta-analysis of European and Japanese studies which made use of objective measures of neighbourhood walkability and walking showed that adults who live in high compared to low walkable neighbourhoods accumulate overall 766 more steps per day [14]. Socio-environmental contexts may modify the neighbourhood walkability-total physical activity relationship. The beneficial role of neighbourhood walkability on physical activity may be smaller in North America than in Europe/Asia, due to sociocultural differences in physical activity preferences and greater reliance on cars in North America [30].

While some previous studies have demonstrated that not having a car [31, 32], is associated with higher levels of total physical activity, we are the first to show that this factor is associated with greater levels of physical activity occurring specifically within home neighbourhoods. Participants who had regular access to a car achieved approximately 613 fewer steps/day in their home neighbourhoods (95 % CI 284 to 942) than those who did not have regular access to a car. This effect is on par with seasonal deficits in daily steps counts that we observed in another cohort of adults with type 2 diabetes living in Montreal [2]. There we found a deficit of 758 steps per day in the fall/winter compared to the spring/summer (95 % CI −1037 to −479). An increase of 613 steps per day represents 12.3 % of this group’s total daily steps (95 % CI 5.7 to 18.9). With the vast majority of our cohort having regular access to a car, reducing reliance on cars may be an effective way of facilitating increases in neighbourhood-based physical activity among adults with type 2 diabetes, an increase that if sufficient in magnitude might actually increase total physical activity. It is important to note, however, that car access was not conclusively associated with overall physical activity in this population.

International evidence points to remarkably consistent lower diabetes incidence in more walkable neighbourhoods [17, 18, 33]. In a study of 214,882 recent immigrants and 1,024,380 long-term residents living in Toronto (Canada) living in less walkable neighbourhoods (based on population density, residential density, street connectivity, and the availability of retail stores and services) was associated with a higher incidence of diabetes after adjustment for age and area-level poverty (Lowest versus highest walkability quintile; Immigrant men: relative risk [RR] 1.58, 95 % CI 1.42 to 1.75, Immigrant women: RR 1.67, 95 % CI 1.48 to 1.88, Long-term resident men: RR 1.32, 95 % CI 1.26 to 1.38, Long-term resident women: RR 1.24, 95 % CI 1.18 to 1.31) [17]. Similarly, in an analysis of 512,061 adults living in Sweden, adults who live in the lowest decile of neighbourhood walkability (based on street connectivity, land use mix, and residential density) were found to have a 33 % higher odds of developing incident type 2 diabetes over four years of follow-up (Odds Ratio (OR): 1.33; 95 % CI 1.13 to 1.55) after adjustment for neighbourhood deprivation [18]. Total physical activity is the presumed link between neighbourhood walkability and diabetes incidence [17, 18]. It is surprising then that we did not observe an association between neighbourhood walkability and total physical activity. There are several possible explanations for this. First, the positive association between neighbourhood walkability and diabetes incidence may be due to unmeasured variables such as the food environment. More walkable neighbourhoods may have a greater availability of healthy food outlets that may reduce the risk of cardiometabolic complications. This theory is supported by our signaled albeit inconclusive finding that participants living in a more walkable neighbourhood may have lower BMIs than participants living in less walkable neighbourhoods, even after adjustment for total physical activity. Another explanation may be confounding by socioeconomic status. In the Toronto-based study, residual confounding was a possibility since area-level poverty was used as a proxy for individual-level income. This is supported by the fact that the association in the Swedish study was attenuated after additional adjustment for individual-level income as well as age, sex, and education (Adjusted OR: 1.16; 95 % CI 1.00 to 1.34) [17]. Lastly, it is possible that our exclusion of a large subset of participants due to insufficient wear-time (i.e., 28.8 %) may have biased our results towards the null. Participants who did not accumulate sufficient wear-time had greater car access (i.e., were likely less active) and lived in less walkable neighbourhoods. Had we been able to include these participants in our analyses a stronger association may have emerged.

There are several strengths to our study. First, we avoided biases arising from participants forgetting to wear one of the devices by using a device that combined an accelerometer and a GPS into one device. This is an improvement over the two previously conducted studies [18], in which participants wore two separate devices. Other strengths include the use of objective measures of walkability and physical activity, and consideration of individual-level covariates and confounders. Some limitations should also be noted. First, our results may not be generalizable to all individuals with type 2 diabetes since only a unique subset of adults with type 2 diabetes may have agreed to participant in this study. Second, 28.8 % of participants did not accumulate enough valid GPS-accelerometer data to be included in the final analyses. Although the mechanism by which these data are missing is unknown, not including these participants in our analyses may have biased our association towards the null as noted above. Third, our sample size limited our ability to assess effect modification. It is possible that factors such as gender or employment status modify the walkability-physical activity relationship. Effect modification by these factors should be assessed in future studies. Lastly, our pedometer-assessed daily steps were not location specific and this means that our daily step estimates represent only approximations of the corresponding changes in VeDBA.

## Conclusions

Our study determined neighbourhood walkability to increase home neighbourhood-based physical activity but no conclusive influence on total activity was discerned in this cohort of adults with type 2 diabetes. Other factors, in combination with walking friendly environments, will need to be leveraged to facilitate meaningful increases in overall PA among adults with diabetes. More research is needed to identify the suite of possible interventions that will facilitate home neighbourhood-based physical activity and ultimately higher levels of overall physical activity.

## Additional files

Additional file 1: Characteristics of the participants that were retained in and excluded from the main analyses. (DOCX 23 kb) Additional file 2: Linear regression estimates for the associations between neighbourhood walkability and total VeDBA accumulated anywhere with corresponding changes in daily steps (*n* = 97). (DOCX 17 kb)

## Abbreviations

BMI: Body mass index
CI: Confidence interval
GIS: Geographic Information System
GPS: Global Positioning System
PA: Physical activity
SD: Standard deviation
SMARTER: Step Monitoring to Improve ARTERial Health
VeDBA: Vector of the Dynamic Body Acceleration
