# Supplementary material for: Neighbourhood walkability and home neighbourhood-based physical activity: an observational study of adults with type 2 diabetes
Source: BMC Public Health. 2016 Sep 9;16(1):957. doi: 10.1186/s12889-016-3603-y (PMC5017036; doi:10.1186/s12889-016-3603-y)
Supplement: Additional file 1: — Characteristics of the participants that were retained in and excluded from the main analyses. (DOCX 23 kb) [file 12889_2016_3603_MOESM1_ESM.docx]

**Additional file 1.** Characteristics of the participants that were retained and excluded from the main analyses.

|  | Retained (n=97) | All excluded (n=59)^a,b^ | Excluded due to missing GPS-accelerometer data (n=45)^c^ |
| --- | --- | --- | --- |
|  | *mean (SD)* | *mean (SD)* | *mean (SD)* |
| Age, *years* | 59.5 (10.5) | 59.9 (10.6) | 60.0 (10.5) |
| Body mass index, *kg/m^2^* | 31.5 (4.5) | 32.1 (5.5) | 32.4 (5.7) |
| Time since diabetes diagnosis, *years* | 10.3 (7.6) | 11.9 (8.8) | 13.1 (9.0) |
| Years at current address | 18.9 (13.9) | 16.5 (12.3) | 16.8 (12.6) |
| Daily steps, *years* | 4,980 (2,798) | 5,357 (2,867) | 4,977 (2,950) |
|  |  |  |  |
| Street connectivity, *number of ≥3 way intersections/km^2^* | 27 (14) | 25 (13) | 26 (12) |
| Land use mix (Score range: 0 to 1) | 0.30 (0.23) | 0.26 (0.17) | 0.26 (0.17) |
| Population density, *population count/km^2^* | 8,915 (8,351) | 7,206 (5,939) | 7,663 (5,879) |
| GIS-derived walkability index | 0.17 (2.29) | -0.25 (1.77) | -0.25 (1.78) |
|  |  |  |  |
|  |  |  |  |
|  | *%* | *%* | *%* |
| Women | 43.3 | 54.2 | 55.6 |
| Married/common-law | 69.1 | 71.4 | 68.4 |
| University education | 53.6 | 37.9 | 40.9 |
| Employed | 61.9 | 53.5 | 53.3 |
| Immigrant | 51.6 | 45.8 | 46.7 |
| Depressed mood | 30.9 | 22.4 | 22.2 |
| Dog ownership | 16.5 | 17.2 | 17.8 |
| Ever smoker | 44.3 | 43.1 | 40.9 |
| Insulin use | 30.9 | 35.6 | 40.0 |
| Car access | 74.2 | 76.8 | 81.8 |
| Spring/summer assessment (*versus* fall/winter) | 40.2 | 32.2 | 26.7 |

^a^ Excluded if participant had insufficient wear-time on their multi-sensor devices, GIS land use data were unavailable for their neighbourhoods, the GPS-accelerometer device malfunctioned, or covariate data were missing.

^b^ Street connectivity and population density (n=43), land use mix (n=41), time since diabetes diagnosis and regular car access (n=56), married/common-law (n=49), depressed mood, years at current address, university, employed, dog ownership and ever smoking (n=58).

^c^ Street connectivity, population density, and land use mix (n=33), married/common-law (n=38), university education, ever smoker, and regular car access (n=44).
