# Supplementary material for: Neighbourhood walkability and home neighbourhood-based physical activity: an observational study of adults with type 2 diabetes
Source: BMC Public Health. 2016 Sep 9;16(1):957. doi: 10.1186/s12889-016-3603-y (PMC5017036; doi:10.1186/s12889-016-3603-y)
Supplement: Additional file 2: — Linear regression estimates for the associations between neighbourhood walkability and total VeDBA accumulated anywhere with corresponding changes in daily steps (n = 97). (DOCX 17 kb) [file 12889_2016_3603_MOESM2_ESM.docx]

**Additional file 2.** Linear regression estimates for the associations between neighbourhood walkability and total VeDBA accumulated anywhere with corresponding changes in daily steps (n=97).

|  | Percent change in one SD of  total VeDBA (95% confidence intervals)^a,b^ | Corresponding change in  daily steps (95% confidence intervals)^c^ |
| --- | --- | --- |
| *Model 1* | 10.6 (-7.4 to 28.6) | 168 (-118 to 454) |
| *Model 2* | 8.6 (-9.3 to 26.4) | 137 (-148 to 419) |
| *Model 3* | 7.2 (-11.4 to 25.7) | 114 (-181 to 408) |
| *Model 4* | -0.6 (-20.1 to 18.9) | -10 (-319 to 300) |
| *Model 5* | 0.7 (-13.7 to 15.2) | 11 (-218 to 242) |

^a^ **Model 1:** Unadjusted. **Model 2:** Adjusted for age, BMI, sex. **Model 3:** Adjusted for age, BMI, sex, university, and season. **Model 4:** Adjusted for age, BMI, sex, university, season, car access and residential self-selection. **Model 5:** Adjusted for age, BMI, sex, university, season, car access, residential self-selection and valid wear-time accumulated anywhere.

^b^ Effect estimates represent the percent change in one standard deviation of total VeDBA (95% confidence interval) within neighbourhoods (excluding homes) for every one-standard deviation increase in the GIS-derived neighbourhood walkability index. Calculated by multiplying the original estimate by the standard deviation of the walkability index (i.e., 2.16), dividing the result by the SD of the outcome (i.e., 240065.36) and multiplying by 100.

^c^ Calculated using the following formula: daily steps=-548+0.0089*total VeDBA occurring anywhere)*(% change in one SD of total VeDBA occurring anywhere/100) where VeDBA occurring anywhere equals one SD of VeDBA occurring anywhere (i.e., 240,065.36)
